# Emerging tools and methods to study cell signalling mediated by branched ubiquitin chains

**DOI:** 10.1042/BST20253015

**Published:** 2025-05-16

**Authors:** Matthew R. McFarland, Yogesh Kulathu

**Affiliations:** MRC Protein Phosphorylation and Ubiquitylation Unit, University of Dundee, Dundee, Scotland, U.K

**Keywords:** activity-based probes, chemical biology, deubiquitinases, proteases, protein engineering, ubiquitin binding domains

## Abstract

Branched ubiquitin chains are complex molecular structures in which two or more ubiquitin moieties are attached to distinct lysine residues of a single ubiquitin molecule within a polyubiquitin chain. These bifurcated architectures significantly expand the signalling capacity of the ubiquitin system. Although branched chains constitute a substantial fraction of cellular polyubiquitin, their biological functions largely remain enigmatic due to their complex nature and the associated technical challenges of studying them. Recent technological innovations have enabled the identification of key molecular players and revealed essential roles for branched chains in diverse cellular processes. In this review, we discuss the bespoke strategies that have driven these discoveries, as well as the technologies needed to advance this rapidly evolving field.

## Introduction

**Figure 1:**
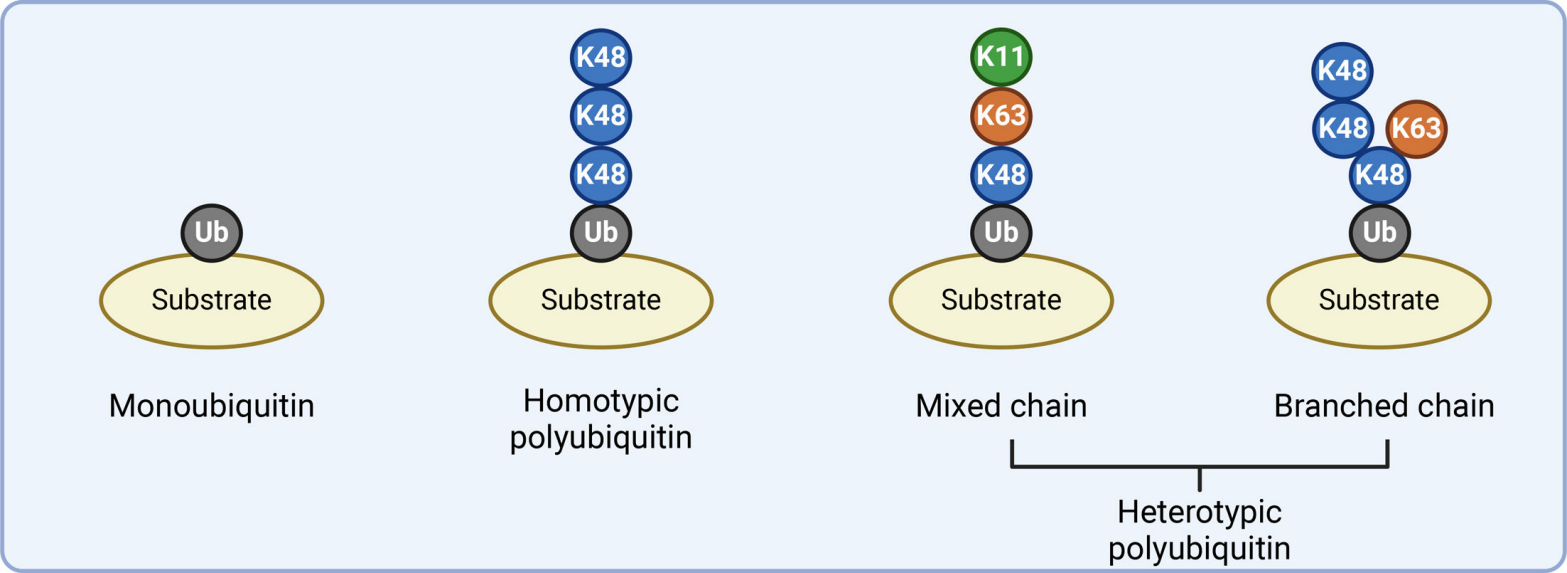
Types of ubiquitin modifications. Grey spheres represent mono- or proximal ubiquitin. Coloured spheres represent distal ubiquitin linked via the indicated residue.

Ubiquitylation is a fundamental posttranslational modification in eukaryotic cell signalling. Through an enzymatic cascade involving ubiquitin-activating E1, ubiquitin-conjugating E2 and E3 ubiquitin ligase enzymes, the C-terminus of ubiquitin is covalently attached to lysine, serine, threonine or N-terminal methionine residues on a substrate protein [1,2]. By virtue of its own seven lysines and N-terminal methionine, ubiquitin itself can act as a substrate, enabling the formation of polyubiquitin chains (Figure 1). These eight distinct linkage types result in chains with unique three-dimensional conformations, creating specific binding surfaces that affect interactions and downstream fate of modified proteins [3].

Polyubiquitin chains can be classified into distinct architectural types based on their linkage pattern. Homotypic chains are polymers in which all constituent ubiquitins are connected through the same lysine residue or N-terminal methionine. In contrast, heterotypic chains incorporate multiple linkage types within a single polymer and can be further subdivided into two categories: mixed and branched chains. Mixed chains contain multiple linkages that may alternate, but each ubiquitin in the chain is modified at only one position. Branched chains represent a more complex architecture where at least one ubiquitin moiety within the chain is modified at two or more positions simultaneously, creating a bifurcation point that gives rise to chain branches (Figure 1).

Theoretically, 28 different trimeric branched ubiquitin chain types containing two different linkages can be formed (e.g. a proximal ubiquitin moiety modified at both K48 and K63). Of these, only three have been identified in cells and linked to biological functions [4–6]. K11-K48 branched chains regulate protein degradation [7–9] and cell cycle progression [10,11]; K29-K48 chains mediate proteasomal degradation [12]; and K48-K63 chains serve multiple functions, including proteasomal degradation [9,13], NF-kB signalling [14] and as a signal for p97/valosin-containing protein (VCP) processing [15]. While M1-K63 [16] and K6-K48 heterotypic ubiquitin [17] have been detected in cells, evidence for their existence specifically within branched architectures remains inconclusive.

The existence of branched ubiquitin chains was first reported approximately two decades ago [18,19]. However, detailed investigation of these complex modifications has been limited by a lack of tools and methodologies to study them. In this review, we highlight recent advances that have enabled the study of branched ubiquitin and discuss the tools and approaches needed to advance future research in this emerging field.

### An intuitive and common nomenclature for branched chains

**Figure 2:**
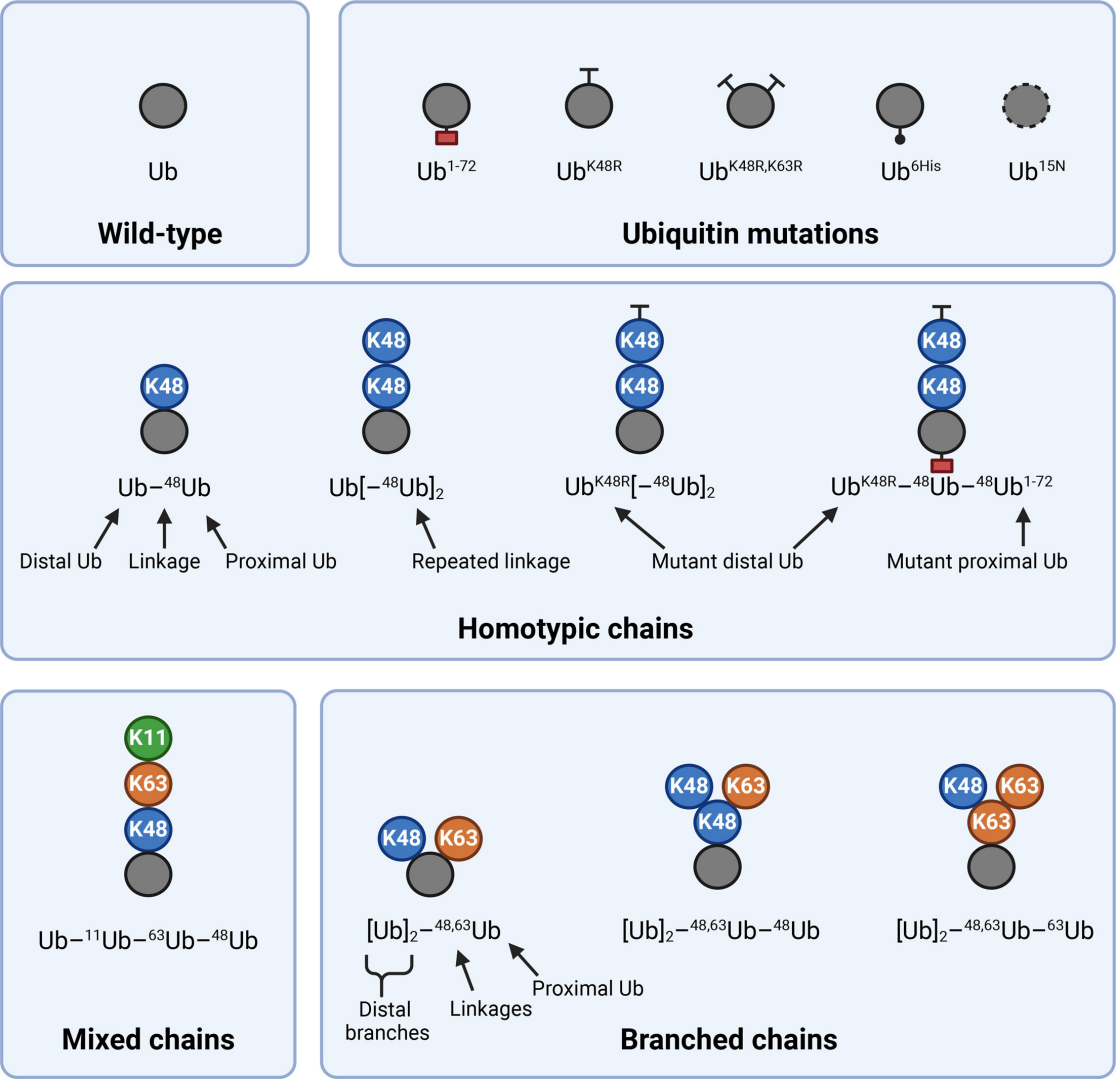
An intuitive nomenclature to describe ubiquitin chains. Grey spheres represent mono- or proximal ubiquitin. Coloured spheres represent distal ubiquitin linked via the indicated residue. In this nomenclature, ubiquitin building blocks are written from distal to proximal, connected by en dashes (–), with the linked residue on the proximal ubiquitin indicated by the preceding superscript. Repeated units can be indicated via square brackets followed by quantity in superscript (e.g. Ub[–^48^Ub]_2_ for a homotypic K48-linked trimer). Mutations or modifications to a particular ubiquitin moiety are indicated in superscript following that ubiquitin (e.g. Ub^1-72^ for a C-terminally truncated ubiquitin). Branches are written in square brackets (e.g. [Ub]_2_–^48,63^Ub describes a branched K48-K63 ubiquitin trimer).

As the branched ubiquitin field grows and develops, a standardised nomenclature to accurately describe these chains is required to avoid confusion and support reproducibility. To this end, we recommend an adapted version of the nomenclature system originally proposed by Fushman and colleagues as illustrated in Figure 2 [15,20].

## Building branched ubiquitin chains

The ability to recombinantly make branched ubiquitin chains of defined linkages and lengths is essential to understand their distinct signalling functions. These chains are invaluable reagents in the identification and characterisation of ubiquitin binding domains, exploring deubiquitinase (DUB) substrate selectivity, investigating recognition and processing by molecular machines such as p97 and the proteasome and developing detection reagents such as antibodies and synthetic binders. With *in vitro* assembly, a range of modifications such as mutations, tags or isotopic labels can be introduced at specific points to functionalise chains for various analyses.

For homotypic chains, well-established enzymatic synthesis methods exist for seven of the eight linkage types using specific combinations of E2/E3 enzymes and DUB treatments [21–25]. In addition, chemical synthesis methods have been developed for all eight linkage types [26,27]. In contrast, the ability to make branched chains of defined architectures has lagged due to our limited knowledge of the cellular enzymes responsible for their assembly.

### Enzymatic branched chain assembly

**Figure 3:**
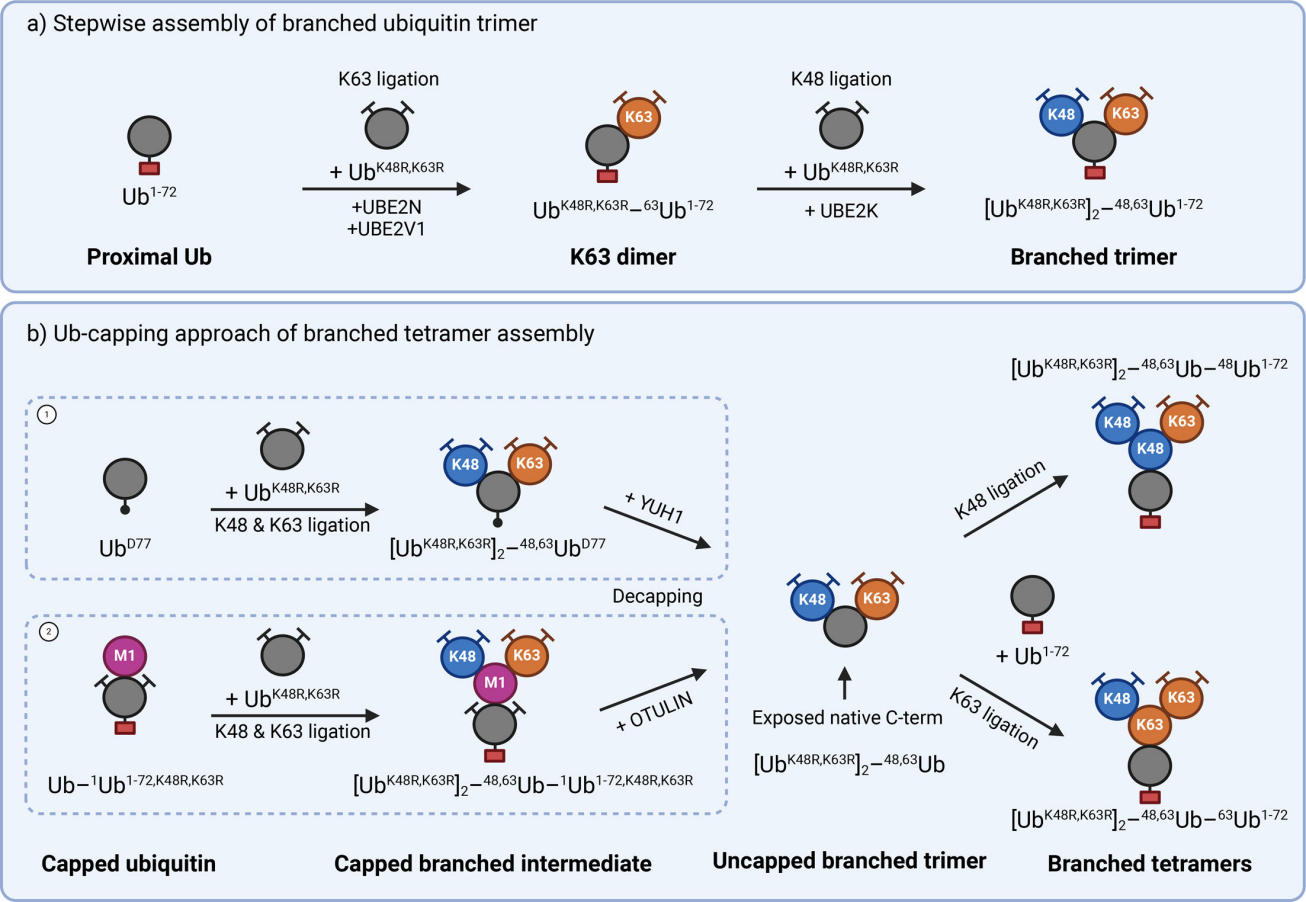
Enzymatic assembly of branched ubiquitin chains. (**A**) Step-wise assembly of a branched ubiquitin trimer. First, K63 dimer is assembled by ligating Ub^K48R,K63R^ onto a C-terminally blocked proximal ubiquitin (e.g. Ub^1-72^) using K63-specific UBE2N/UBE2V1. A second Ub^K48R,K63R^ is then added by K48-specific UBE2K to produce a branched K48-K63 trimer. (**B**) Assembly of branched ubiquitin tetramers using capped ubiquitin. Starting with Ub^D77^ or M1-linked dimer, a K48 and K63 branched intermediate is produced by ligation of Ub^K48R,K63R^ in a step-wise manner as in A. Intermediate is decapped with YUH1 (for Ub^D77^) or OTULIN (for M1 cap) to expose a native C-terminus on the proximal ubiquitin for subsequent chain extension.

While several E3 ligases, including UBE3C, UBR5 and cIAP1, can generate branched ubiquitin chains [9,10,28], they have limited use in assembling defined branched architectures. Consequently, current methods rely on combinations of ubiquitin mutants to systematically make branched chains. The method of choice for generating branched ubiquitin trimers has been to start with a C-terminally truncated (Ub^1-72^) or blocked (e.g. Ub^D77^ or Ub^6his^) proximal ubiquitin. Mutant distal ubiquitins are then ligated sequentially using specific enzymes for each individual linkage [10,20,29–31] (Figure 3A).

This approach enables straightforward and reliable assembly of branched trimers of various linkages using established protocols. For example, branched K48-K63 trimers can be formed by first generating a K63 dimer from Ub^1-72^ and Ub^K48R,K63R^ using UBE2N and UBE2V1, followed by K48 linkage of Ub^K48R,K63R^ to the proximal Ub^1-72^ using a K48-specific enzyme such as UBE2R1 or UBE2K. Alternatively, the yeast homologue of UBE2K, Ubc1, is able to assemble K48 branches on K63-linked chains [31,32].

A drawback of this approach is that the modified C-terminus of the proximal ubiquitin prevents further extension of the chain. To enable the assembly of more complex tetrameric branched ubiquitin structures, our lab recently adapted the previously described Ub-capping approach that uses the yeast DUB Yuh1 to trim the C-terminus of a D77-blocked ubiquitin [15,33]. We initiated assembly of K48-K63 branched chains with an M1-linked dimer comprising a wildtype distal ubiquitin and a proximal Ub^1-72, K48R, K63R^ mutant. Following K48 and K63 ligation to the distal ubiquitin, the M1-specific DUB OTULIN removes the proximal cap (Figure 3B), exposing the native C-terminus of the branch point ubiquitin to facilitate further chain extension. Through use of a universal M1 dimer where all lysines on the proximal ubiquitin bar K33 have been mutated to arginine (Ub[–^1^Ub^KallR but K33^]), this approach can be extended to build other branched chain types. As OTULIN requires K33 on the proximal ubiquitin for cleavage [15], the generation of branched chains containing these linkages would require an alternative strategy.

Recently, a photo-controlled enzymatic assembly method was developed by Furuhata and colleagues, which uses chemically synthesised ubiquitin moieties (discussed in a later section) where target lysine residues are protected by photolabile 6-nitroveratryloxycarbonyl (NVOC) groups [34]. Through alternating cycles of K63-specific elongation, the deprotection of the NVOC group with UV irradiation and K48-specific elongation, they demonstrated assembly of a K48-K63 branched tetramer. This approach offers the advantage of making branched chains using wildtype ubiquitin.

Ubiquitin chains can also be assembled independently of the conventional E1-E2-E3 pathway, either through the use of alternative enzymes or by chemical means, and various studies have begun to apply these methodologies to branched polyubiquitin [35–38]. The Strieter lab, for example, has used a thiol-ene coupling technique to produce homotypic and branched ubiquitin trimers [39]. This approach modifies the distal ubiquitin C-terminus with allylamine for reaction with proximal ubiquitin containing lysine-to-cysteine mutations at desired branch points. The resulting chains contain near-native isopeptide linkages that can still be cleaved by linkage-specific DUBs [30,39,40].

### Chemical synthesis of ubiquitin chains

Chemical synthesis offers a powerful alternative to biosynthetic approaches for generating ubiquitin chains. Ubiquitin can be fully chemically synthesised through linear solid phase peptide synthesis (SPPS) [26] or by native chemical ligation (NCL) of SPPS-generated fragments [27]. A key advantage of chemical synthesis is the ability to incorporate diverse modifications including mutations, tags, warheads and other functional groups that would be challenging or impossible to incorporate through conventional biosynthesis [41]. The strategic placement of reaction handles at specific acceptor target lysine sites and donor ubiquitin C-terminus enables subsequent assembly of the synthesised monomers into chains via any of the eight homotypic linkages [26,27].

In addition to homotypic chains, branched ubiquitin chains have been successfully produced via full chemical synthesis. For example, branched K11-K48 ubiquitin of varying lengths has been generated utilising an innovative ‘isoUb’ core strategy. This synthesised core consists of residues 46–76 of the distal ubiquitin linked via a pre-formed isopeptide bond of the desired linkage to residues 1–45 of the proximal ubiquitin. The core additionally contains an N-terminal cysteine and C-terminal hydrazide, enabling efficient NCL of additional ubiquitin building blocks [42].

### Exploiting genetic code expansion to assemble ubiquitin chains

Genetic code expansion enables complete chemical synthesis for generating functionalised ubiquitin chains. This approach uses the site-specific incorporation of noncanonical amino acids through repurposing of the amber stop codon (UAG) in *E. coli* with an orthogonal tRNA/tRNA synthetase pair to functionalise ubiquitin monomers for precise chain assembly. Reviewed in detail in [43].

The Fushman lab utilised this approach to synthesise K11-K33 branched trimers ([Ub]_2_-^11,33^Ub) by incorporating butoxycarbonyl (BOC) lysine at positions K11 and K33 through amber suppression [44]. The ε amino group of this lysine derivative is protected from ubiquitin modification by the presence of a *tert*-BOC group. This method subsequently involves allyloxycarbonyl (Alloc) protection of the remaining lysines, BOC deprotection and silver-mediated chemical ligation for branched trimer assembly, followed by Alloc deprotection, refolding and purification.

Genetic code expansion has also enabled branched ubiquitin assembly through click chemistry, producing non-hydrolysable chains resistant to DUB activity [45]. This approach combines a proximal ubiquitin containing lysine-to-cysteine mutations modified with propargyl acrylate and a distal ubiquitin incorporating the methionine analogue azidohomoalanine (Aha) at its C-terminus. These modified monomers undergo click reactions to generate various branched architectures, depending on the position of the propargyl acrylate.

The Lang laboratory developed a hybrid approach combining sortase enzymes with genetic code expansion to assemble complex ubiquitin and ubiquitin-like modifier (UBL) polymers [37]. Their method relies on the incorporation of the noncanonical amino acid N^6^-((2-azidoacetyl)glycyl)-L-lysine (AzGGK) at desired modification sites in the proximal ubiquitin through amber suppression. These modified ubiquitins are then combined with distal ubiquitin bearing a C-terminal recognition sequence for the engineered sortase Srt2A. Branched ubiquitin chains can be formed using this method through the incorporation of two or more AzGGK modifications. An advantage of this approach is that the resulting chains are linked via an isopeptide bond, and there is no need for harsh chemical deprotection and refolding. Additionally, the linked ubiquitins will have modified C-termini due to the inclusion of the sortase recognition motifs, making them resistant to DUB activity. However, these modifications may affect downstream interactions that rely on the C-terminus of ubiquitin. Notably, this method allows subsequent site-specific ubiquitylation of AzGGK-modified substrate protein with branched or unbranched chains using a second sortase, Srt5m [37].

## Use of branched chains to reveal molecular players and mechanisms

*In vitro* assembled branched ubiquitin chains serve as valuable reagents for investigating cellular functions and identifying molecular interaction partners.

### Identifying readers

Knowing the identity of proteins and domains that interact with branched ubiquitin chains provides critical insights into their cellular roles. Affinity purification using immobilised branched chains as bait, followed by mass spectrometry (MS), can reveal how ubiquitin chains are decoded [46]. Recent studies used this approach with great success to reveal the existence of specific binders of branched chains [15,31] (Table 1). Interactors of K48-K63 branched chains discovered through these studies include proteins modulating histone modifications (USP15, MORC3), DNA replication (RFC1), endocytosis (HIP1), protein quality control (UBR4, USP13, DNAJB2) and p97-dependent processing (ATXN3, ZFAND2B, RHBDD1) [10,48,50,52,54,57,58,64,65,67]. The enrichment of proteins involved in protein quality control and p97-dependent processing aligns with previous findings suggesting that branched chains may function as priority signals for processing problematic or time critical substrates [7,8,10,13,69]. These discoveries highlight the potential of extending similar analyses to identify specific ‘reader’ modules for other branched ubiquitin chain architectures.

**Table 1:** Readers and deubiquitinases selective for branched ubiquitin chains.

Readers	Cellular function	Ubiquitin-binding domains	Refs
ANKFY1	Endosomal transport		[31,47]
ATXN3	Deubiquitinase, DNA repair	UIM x 3	[15,48]
BMERB1	Unknown		[31]
CAPN15	Calpain peptidase		[15,49]
DNAJB2	Co-chaperone	UIM x 2	[15,50]
GGA3	Endosomal transport	GAT	[31,51]
HIP1	Endocytosis	ANTH	[31,52]
MIB2	E3 ligase		[31,53]
MORC3	Histone regulation		[15,31,54]
PARP10	ADP ribosyltransferase, DNA repair	UIM x 2	[31,55]
PRKCZ	Protein kinase		[15,56]
RFC1	DNA replication	MIU-like	[15,31,57]
RHBDD1	Serine peptidase	UIM	[15,58]
RIOK3	Protein kinase, RQC	MIU	[15,59,60]
ROCK2	Protein kinase	Possible UIM x 2	[15,61]
SYN2	Neurotransmitter secretion		[15,62]
TAPBP	Peptide antigen loading		[15,63]
UBR4	E3 ligase		[10,31]
USP13	Deubiquitinase, ERAD	UBA x 2, UBP-type ZnF	[15,64]
USP15	Deubiquitinase, histone regulation		[15,65]
USP48	Deubiquitinase, DNA repair	DUSP x 3, UBL	[31,66]
ZFAND2B	ERAD	UIM x 2	[15,67]
ZFAND5	Proteasomal activator	A20-type ZnF	[15,68]
**DUBs**	**Cellular function**	**Linkage preference**	**Refs**
ATXN3	p97-associated DUB	K63 branches	[15]
MINDY1	Unknown	K48 branches	[15]
MINDY3	Unknown	K48 branches	[15]
UCHL5	Proteasome-associated DUB	K48 branches	[15,29,30]

### Studying debranching DUBs

DUBs regulate ubiquitin signalling by removing ubiquitin from substrates or trimming chains to both fine-tune and shut-off signals. Humans have approximately 100 DUBs that are distributed across seven families: six cysteine protease families (USP, MJD, OTU, UCH, MINDY, ZUP) and a sole metalloprotease family (JAMM) [70]. DUBs have various substrate preferences, including linkage type and chain length [71]. For example, MINDY family DUBs are highly specific for K48 linkages, while the USP family tends to be linkage-agnostic and cleave a wide array of chain types [72–74].

Typically, the activity and linkage preference of a DUB are assayed in a qualitative manner by monitoring cleavage of polyubiquitin chains [75]. While this works well for simple homotypic substrates, a major limitation of these assays is that they cannot identify which specific linkages are cleaved. Hence, they have limited utility to study DUB activity against complex architectures such as branched ubiquitin. Several innovative approaches have recently emerged to address this limitation.

**Figure 4:**
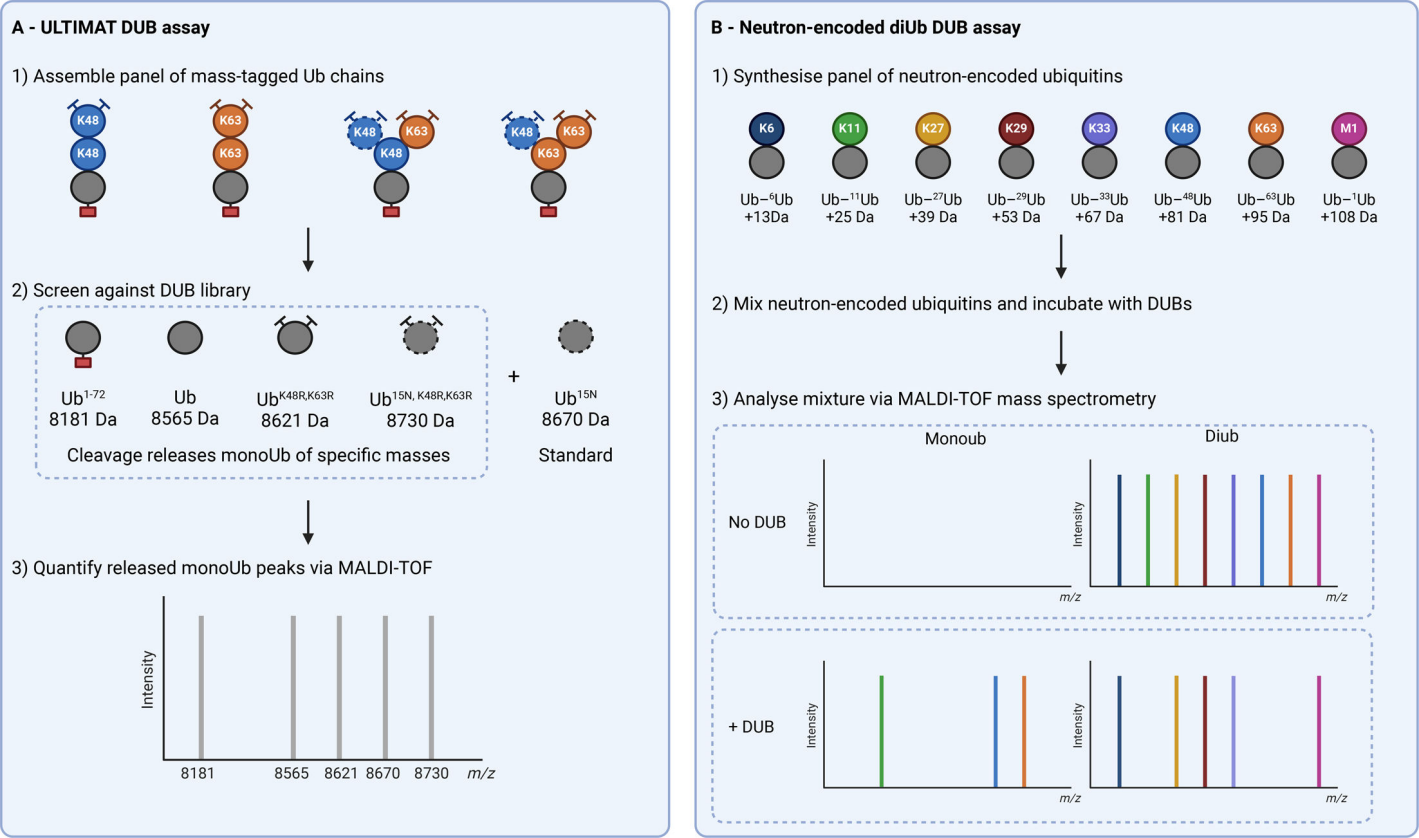
Identifying debranching DUBs with mass spectrometry. (**A**) The ULTIMAT DUB assay. Substrate chains are assembled with each ubiquitin monomer having a unique mass (e.g. through point mutation or incorporation of heavy isotopes). DUB cleavage releases monoUb of specific masses, allowing identification of cleaved linkages via MALDI-TOF-MS. (**B**) Neutron-encoded diUb assay. Substrate chains are chemically synthesised and isotopically labelled, so that each linkage type has a unique mass. Chains are mixed and incubated with DUB of interest and subsequently analysed via intact MS. DUB cleavage of a particular linkage will reduce the intensity of its diUb peak and cause an appearance of the corresponding monoUb peak. DUB, deubiquitinase; ULTIMAT, Ubiquitin Linkage Target Identification by Mass Tagging.

Our laboratory recently developed the Ubiquitin Linkage Target Identification by Mass Tagging (ULTIMAT) DUB assay [15]. This method utilises substrate chains where each of the ubiquitin moieties has a defined mass. Once incubated with the DUB of interest, the sample is analysed via MALDI-TOF MS with the released ubiquitin species indicating the cleaved linkages (Figure 4A). This method identified MINDY-1, MINDY-3 and ATXN3 as debranching enzymes targeting K48 and K63 linkages in branched chains [72,76] (Table 1). While this assay has successfully been used in identifying debranching DUBs, one limitation is that the use of ubiquitin point mutants to make the substrate ubiquitin chains can affect DUB activity in rare cases . This was seen with USP5 which can readily cleave K63 chains assembled from wildtype ubiquitin but failed to cleave the mutant substrate chains in the ULTIMAT assay [15].

Another limitation of conventional DUB assays is that they do not consider that, in cells, different ubiquitin linkages will co-exist and can compete for DUB binding, with each enzyme perhaps exhibiting substrate preferences that would not be apparent in a monoplex assay. A recent study tackled this problem by combining chemical synthesis and heavy amino acid labelling to assemble a complete panel of homotypic diubiquitin chains with distinct masses, allowing multiplexing and subsequent deconvolution of each via MS, providing a more accurate profile of an enzyme’s specificity [77] (Figure 4B). While this method has only been applied to diubiquitin thus far, this approach could potentially be extended to branched chains, provided that sufficient labels can be incorporated to allow mass discrimination between them.

In an alternative approach, the Strieter lab recently utilised ion-mobility mass spectrometry (IM-MS) as a means to determine DUB specificity against complex substrate mixtures [78]. This approach relies on the fact that ubiquitin chains of different linkages and topologies possess unique collision-induced unfolding (CIU) fingerprints. This strategy can, therefore, be used to identify the substrate preference of a particular DUB by incubating it with a mixture of chains and deconvoluting the resulting CIU fingerprints, with any absent fingerprints corresponding to the linkages targeted by that enzyme. Using this method, the authors confirmed a strong preference of the proteasome-associated debranching DUB UCHL5 for K6-K48 branched ubiquitin [29,30] (Table 1).

Activity-based ubiquitin probes featuring C-terminal reactive warheads are useful tools for identifying and profiling DUBs [79,80]. The development of di- and triubiquitin probes permit the study of both linkage and length preference on enzyme activity [81,82]. Recent studies have sought to expand this suite of tools to branched ubiquitin with probes assembled from a subset of K6, K11, K48 and K63 branched linkages [83,84].

## Detection of branched ubiquitin chains in cells

Perhaps the biggest challenge for branched ubiquitin research has been identifying these polymers in cells. Similar to the identification of DUBs with debranching activity, MS-based approaches have so far led the way in this regard.

### Mass spectrometry approaches

**Figure 5:**
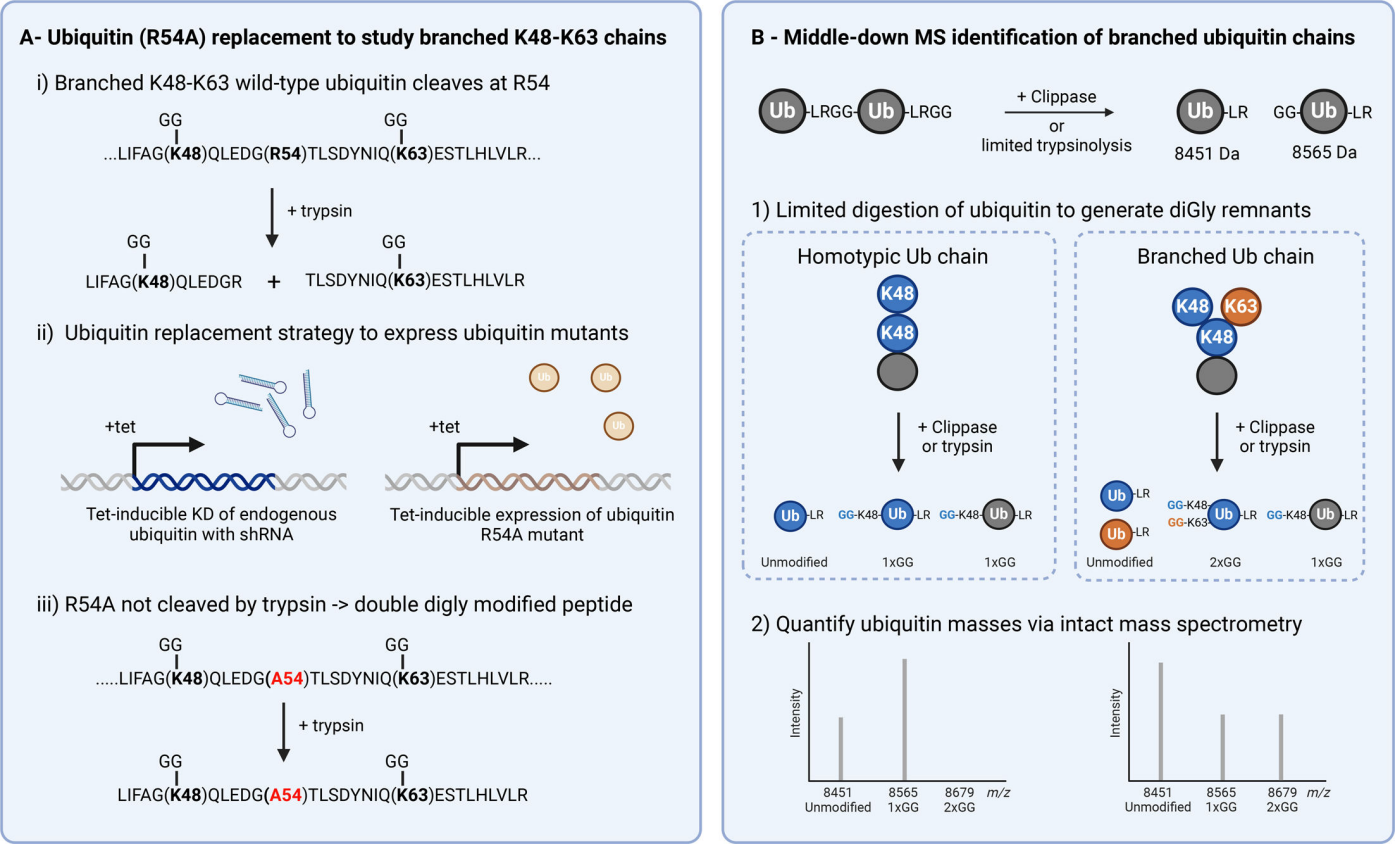
Detecting branched ubiquitin chain in cells. (**A**) Ubiquitin (R54A) replacement to study branched K48-K63 chains. Tryptic digestion of K48-K63 branched ubiquitin will produce two separate diGly-remnant peptides due to cleavage at R54. The ubiquitin replacement strategy involves simultaneous tetracycline-inducible shRNA knockdown of endogenous ubiquitin alongside rescue with a shRNA-resistant ubiquitin construct bearing mutations of interest. Expression of ubiquitin (R54A) prevents cleavage at that site and allows detection of a double diGly-modified branched peptide. (**B**) Middle-down MS identification of branched ubiquitin chains. Limited trypsinolysis or Ub clippase activity leaves diGly remnants on proximal ubiquitin moieties. Homotypic chains will produce only unmodified and single diGly-modified species, while branched chains will also yield ubiquitins with two or more diGly remnants. The 114 Da increase in mass for each additional diGly allows chain topology to be analysed via intact mass spectrometry.

Tryptic digestion of ubiquitin chains leaves a characteristic di-Glycine (diGly) remnant on the linkage site residue. Branch point ubiquitins that have been modified at multiple sites would, therefore, possess multiple diGly remnants. In the case of K48-K63 branched ubiquitin, tryptic cleavage at R54 would typically produce two separate diGly-modified peptides, hence losing information on whether the diGly remnant came from a branched chain or homotypic chain. To overcome this limitation, the Tanaka and Ohtake labs used a ubiquitin replacement strategy [85], depleting endogenous ubiquitin and replacing it with an R54A-mutant variant, to enable the detection of doubly diGly-modified branched ubiquitin peptides [14] (Figure 5A). This approach revealed that approximately 20% of K63 linkages exist within K48-K63 branched architectures. Further investigation found that these chains are formed in response to IL-1β stimulation through the coordinated action of HUWE1 and TRAF6 E3 ligases, inhibiting the K63 DUB activity of CYLD and promoting NF-kB signalling. However, this method cannot be readily applied to other branched ubiquitin types (K11-K48 and K29-K48) as multiple lysine and arginine point mutations have to be introduced into ubiquitin to prevent tryptic digestion and produce relevant doubly diGly-modified peptides.

Other groups have exploited the 114 Da increase from each diGly remnant to distinguish branched species from monoubiquitylated and unmodified ubiquitin via MS. UbiChEM-MS [11,86] and Ub-clipping [87] are two methods that use middle-down MS to accomplish this. These approaches utilise limited trypsinolysis, or an engineered clippase Lb^pro*^, respectively, to cleave ubiquitin between R74 and G75 and generate truncated diGly-modified ubiquitin for MS analysis (Figure 5B). When combined with tandem ubiquitin binding entity (TUBE) or UBD pull-down from cell lysates to enrich for ubiquitin chains, these approaches found that doubly diGly-modified ubiquitin comprises 4–7% of polyubiquitin. The Strieter lab further utilised a K11-specific antibody to enrich that linkage prior to UbiChEM-MS and demonstrated an accumulation of branched K11-K48 ubiquitin chains following release from mitotic arrest, supporting previous evidence for their role in cell cycle regulation [7,11]. The development of better and more efficient ubiquitin clippases that similarly cleave between R74 and G75, such as the bacterial clippases discovered by the Hoffmann lab [88], offers several advantages over Lb^pro*^ and trypsin-based strategies. These include increased specificity towards ubiquitin and increased clippase activity. In addition, one of the identified family members, PcJOS, exhibited M1-specific cleavage activity, raising the possibility of engineering a suite of ubiquitin-linkage-specific clippase tools.

### Antibodies and nanobodies

Beyond MS-based approaches, specific affinity reagents are essential tools to detect and study branched ubiquitin chains in cellular contexts.

#### Bispecific antibodies

The Rape lab engineered the first bispecific K11-K48 antibody using a previously described heterodimerisation approach to combine K11 and K48 monoclonal antibodies. In this bispecific antibody, one arm binds specifically to K11 and the other to K48, allowing detection of branched ubiquitin species containing both linkages [10]. This reagent has enabled profiling of the formation of branched K11-K48 ubiquitin in cells through Western blotting, immunoprecipitation, MS and immunofluorescence imaging approaches. These experiments confirmed APC/C-mediated assembly of branched K11/K48 [7,11] and revealed that UBR4 and UBR5 also generate these chains in response to proteotoxic stress. Notably, different enzymes create different branched chain architectures. While APC/C builds blocks of K11 on a K48 trunk in collaboration with UBE2S, the chains assembled by UBR4 and UBR5 appear to adopt a different architecture with blocks of K48 branching off a K11 chain. This raises an interesting question as to whether the identity of the ‘trunk’ and ‘branch’ of the chain (i.e. a K11 branch on a K48 chain, and vice versa) affects signalling outcomes. Linkage-specific monoclonal antibodies have been developed against four of the eight homotypic chain types: K11, K48, K63 and M1 [89–91], and these could be leveraged to make bispecific antibodies to any combination of these linkages using a similar strategy.

#### Specific nanobodies

While the K11-K48 bispecific antibody has proven useful for elucidating the roles of these particular chains, to date, it remains the only branched chain type with such a tool. In recent years, nanobodies have been identified as an alternative to conventional antibodies. These consist of the isolated variable domain from camelid single-chain antibodies and, unlike conventional antibodies which require correct heavy/light chain pairing, are sufficient for full antigen binding [92]. Recombinant nanobodies express well in both bacterial and eukaryotic systems, allowing them to be purified at large scale for *in vitro* and structural biology or as intracellular tools for cell biology.

Utilising a synthetic yeast surface-display library [93], our laboratory has recently isolated and matured a highly specific nanobody against K48-K63 branched ubiquitin. This nanobody, NbSL3.3Q, demonstrates sub-nanomolar binding affinity for K48-K63 ubiquitin, which is ~2,500 and ~10,000 times stronger than for unbranched K48 and K63 chains, respectively. Immobilising NbSL3.3Q on beads and incubating cell lysates treated with a panel of inhibitors, we found that this branched ubiquitin species accumulated in cells in response to p97 inhibition, and this has since been replicated by others [94]. Intriguingly, when expressed in cells, we found that a GFP-tagged version of the nanobody localised to sites of laser-induced DNA damage and that this could be enhanced by pre-treating cells with a p97 inhibitor. Together, our results suggest that K48-K63 branched ubiquitin chains act as signals for p97, targeting it to particular substrates for downstream processing.

## Future prospects: seeing the wood for the branches

Of the 28 possible bifurcated branched triubiquitin species, only three have been definitively identified in cells and, while great progress has been made over the past few years in developing tools and methods to facilitate their study, there remain many unanswered questions in the field. Possibly the largest knowledge gap is in how branched ubiquitin is formed in cells. Although some individual enzymes capable of branched chain assembly have been identified [9,10,14,32], we lack methods to systematically screen and identify E2 and E3 enzymes with branching activity. Furthermore, it remains unclear how these enzymes achieve branching activity, and how this activity is regulated.

Identifying the molecular players mediating branched ubiquitin formation, decoding and removal are required to advance the field. Development of detection reagents for other branch types, along with improved and facile MS methods, will be essential to map the cellular landscape of branched ubiquitin. While several approaches exist for synthesising branched chains *in vitro*, we need more efficient methods to generate longer branched chains and incorporate specific probes. Moreover, we currently have limited means to precisely label model substrates with complex branched ubiquitin chains. Such technical advances will be invaluable, for example, to investigate how different branched architectures enable recognition and processing by the proteasome, p97 complexes and debranching DUBs.

Recent studies suggest that branched chains may serve as priority signals for critical cellular processes, including cell cycle regulation, DNA damage responses and protein quality control. Hence, understanding branched ubiquitin chain biology has important implications for human health and therapeutic approaches, which depend on continued innovation to develop better tools and methodology to study these complex modifications.

**Perspectives**

- Branched ubiquitin chains are the result of a ubiquitin moiety becoming modified at two or more linkage sites, producing forks within the chain that have novel surfaces for protein interaction over homotypic linkages. A small number of branched chain types have been identified in cells; however, detailed study of these chain architectures has been limited by a lack of available tools and methodologies.
- Current knowledge suggests that branched chains act as priority signals in diverse cellular processes such as DNA repair, cell cyle regulation and protein quality control. Mass spectrometry-based methods and linkage-specific antibodies and nanobodies have allowed profiling of a subset of branched ubiquitin types, identifying reader and eraser proteins with a preference for branched chains.
- Developing methods and tools to identify the writers (E2s/E3s), readers and erasers of different branched ubiquitin architectures, and expanding the repertoire of linkage-specific detection reagents will be invaluable to further our understanding of how, when and under what cellular circumstances these complex ubiquitin chains are formed.

## Abbreviations

ANTH: AP180 N-terminal homology
Aha: azidohomoalanine
Alloc: allyloxycarbonyl
AzGGK: N^6^-((2-azidoacetyl)glycyl)-L-lysine
BOC: butoxycarbonyl
CIU: collision-induced unfolding
DUB: deubiquitinase
DUSP: domain present in ubiquitin-specific protease
DiGly: diglycine
GAT: GGA and TOM1
IM-MS: ion-mobility mass spectrometry
MALDI-TOF: Matrix-Assisted Laser Desorption/Ionisation Time-of-Flight
MIU: motif interacting with ubiquitin
MS: mass spectrometry
NCL: native chemical ligation
NVOC: 6-nitroveratryloxycarbonyl
SPPS: solid phase peptide synthesis
TUBE: tandem ubiquitin binding entity
UBA: ubiquitin-associated domain
UBL: Ubiquitin-like
UIM: ubiquitin-interacting motif
ULTIMAT: Ubiquitin Linkage Target Identification by Mass Tagging
Ub: Ubiquitin
UbiChEM-MS: Ubiquitin Chain Enrichment Middle-Down Mass Spectrometry
VCP: valosin-containing protein
ZnF: zinc finger
